# T-Cell Responses Are Associated with Survival in Acute Melioidosis Patients

**DOI:** 10.1371/journal.pntd.0004152

**Published:** 2015-10-23

**Authors:** Kemajittra Jenjaroen, Suchintana Chumseng, Manutsanun Sumonwiriya, Pitchayanant Ariyaprasert, Narisara Chantratita, Piyanate Sunyakumthorn, Maliwan Hongsuwan, Vanaporn Wuthiekanun, Helen A. Fletcher, Prapit Teparrukkul, Direk Limmathurotsakul, Nicholas P. J. Day, Susanna J. Dunachie

**Affiliations:** 1 Mahidol-Oxford Tropical Medicine Research Unit, Mahidol University, Bangkok, Thailand; 2 Department of Microbiology and Immunology, Faculty of Tropical Medicine, Mahidol University, Bangkok, Thailand; 3 London School of Hygiene and Tropical Medicine, London, United Kingdom; 4 Sappasithiprasong Hospital, Ubon Ratchathani, Thailand; 5 Department of Tropical Hygiene, Faculty of Tropical Medicine, Mahidol University, Bangkok, Thailand; 6 Centre for Tropical Medicine, University of Oxford, Oxford, United Kingdom; University of California San Diego School of Medicine, UNITED STATES

## Abstract

**Background:**

Melioidosis is an increasingly recognised cause of sepsis and death across South East Asia and Northern Australia, caused by the bacterium *Burkholderia pseudomallei*. Risk factors include diabetes, alcoholism and renal disease, and a vaccine targeting at-risk populations is urgently required. A better understanding of the protective immune response in naturally infected patients is essential for vaccine design.

**Methods:**

We conducted a longitudinal clinical and immunological study of 200 patients with melioidosis on admission, 12 weeks (n = 113) and 52 weeks (n = 65) later. Responses to whole killed *B*. *pseudomallei* were measured in peripheral blood mononuclear cells (PBMC) by interferon-gamma (IFN-γ) ELIspot assay and flow cytometry and compared to those of control subjects in the region with diabetes (n = 45) and without diabetes (n = 43).

**Results:**

We demonstrated strong CD4+ and CD8+ responses to *B*. *pseudomallei* during acute disease, 12 weeks and 52 weeks later. 28-day mortality was 26% for melioidosis patients, and *B*. *pseudomallei-*specific cellular responses in fatal cases (mean 98 IFN-γ cells per million PBMC) were significantly lower than those in the survivors (mean 142 IFN-γ cells per million PBMC) in a multivariable logistic regression model (*P* = 0.01). A J-shaped curve association between circulating neutrophil count and mortality was seen with an optimal count of 4000 to 8000 neutrophils/μl.

Melioidosis patients with known diabetes had poor diabetic control (median glycated haemoglobin HbA_1c_ 10.2%, interquartile range 9.2–13.1) and showed a stunted *B*. *pseudomallei-*specific cellular response during acute illness compared to those without diabetes.

**Conclusions:**

The results demonstrate the role of both CD4+ and CD8+ T-cells in protection against melioidosis, and an interaction between diabetes and cellular responses. This supports development of vaccine strategies that induce strong T-cell responses for the control of intracellular pathogens such as *B*. *pseudomallei*.

## Author Summary

Melioidosis is a key cause of death in South East Asia and Northern Australia. It is caused by the soil-dwelling bacteria *Burkholderia pseudomallei*, and presents as a range of clinical illnesses including pneumonia and bloodstream infections. About two-thirds of patients with melioidosis in Thailand have diabetes, but the immune responses associated with death and diabetes are unknown. This study examined the relationship between immune responses to the bacteria and death by studying the bacteria-specific lymphocyte responses in 200 patients admitted to hospital with acute melioidosis and following the patients for up to one year where possible. 26% of patients died within 28 days despite receiving antibiotics and supportive care. We showed people with melioidosis make bacteria-specific lymphocyte responses, with lower levels seen in fatal cases compared to survivors. People with diabetes make lower responses than people without diabetes, and higher levels of circulating neutrophils on admission to hospital were associated with lower lymphocyte responses both during illness and three months later in survivors. This highlighting of lymphocyte responses to melioidosis is important for the design of vaccines to target at risk groups.

## Introduction

Melioidosis is a severe and often fatal disease common in Southeast Asia and Northern Australia, caused by the soil-dwelling bacterium *Burkholderia pseudomallei*. The main routes of infection are skin inoculation, inhalation, and ingestion of the organism from the environment [1]. The known risk factors are diabetes, alcoholism, renal disease and increasing age, with HIV not a major risk factor [2]. There is a range of presentations including pneumonia, liver and splenic abscesses and septic shock, and the mortality in North-East Thailand is around 40% despite appropriate antibiotic therapy [3]. Melioidosis is now recognised as endemic in an expanding number of countries [4]. The prevalence of diabetes in the region is growing, with the reported Thai prevalence now 6.9% (chiefly Type 2) [5]. Meanwhile changing demographics in the region leave an increasingly ageing population exposed to the bacterium by farming as younger family members migrate to urban regions [6]. A vaccine is therefore in demand and cost effective if targeted towards at-risk groups [7].

The human immune response to melioidosis is far from fully understood. Important roles for Toll-like receptor (TLR) [8–10], and nucleotide-binding oligomerization domain 2 (NOD2) [11] variants, macrophage function [12] and neutrophil response in diabetic patients to *B*. *pseudomallei* [13,14] have been reported. Up to 80% of people in endemic regions have serological responses to *B*. *pseudomallei* whether they have a history of disease or not [15,16].

As an intracellular pathogen [17] capable of chronic disease and latency, cellular adaptive immunity seems likely to be important. There are similarities in the host response to *B*. *pseudomallei* and to *M*. *tuberculosis*, with both being intracellular, sharing the risk factor of diabetes [18] and showing similar gene expression profiles dominated by interferon-mediated signalling pathways [19]. Further support for cellular immunity comes from reports of individuals with HLA-DRB1*1602 in Thailand being at increased risk of melioidosis [20]. Murine models have demonstrated the importance of IFN-γ in survival from experimental challenge [21] and production of IFN-γ in response to *B*. *pseudomallei* by NK (Natural Killer) cells, CD8+ T-cells and CD4+ T-cells [22]. In humans, an association between survival from melioidosis and IFN-γ levels and linked cytokines has been shown [23–25]. Cell mediated immunity has been demonstrated in Australian patients recovered from melioidosis [26]. Memory T-cell responses to *B*. *pseudomallei* in healthy blood donors in an endemic region and some subjects with previous melioidosis have been characterised [27]. However the relationship between cellular responses and survival has not previously been characterised during acute melioidosis.

Vaccine design requires knowledge of naturally acquired immunity, to understand which bacterial antigens are immunogenic and which components of the immune response are protective. The development of vaccines against melioidosis is hindered by knowledge gaps [28]. The aim of this study was to see if the cellular responses to *B*. *pseudomallei* were related to survival in patients with melioidosis, and to see the dynamics of the cellular response over one year in survivors from melioidosis. This work demonstrates an association between lower CD4+ and CD8+ T-cell responses and mortality from the disease, and shows impaired cellular immunity in diabetic patients acutely unwell with melioidosis compared to non-diabetic patients. These findings support an important contribution to control of melioidosis in humans by cellular immunity.

## Materials and Methods

### Ethical approval

The study was approved by the ethics committees of Faculty of Tropical Medicine, Mahidol University (Submission number TMEC 12–014), of Sappasitthiprasong Hospital, Ubon Ratchathani (reference 018/2555) and the Oxford Tropical Research Ethics Committee (reference 64–11). The study was conducted according to the principles of the Declaration of Helsinki (2008) and the International Conference on Harmonization (ICH) Good Clinical Practice (GCP) guidelines. Written informed consent was obtained for all patients enrolled in the study.

### Recruitment of subjects

In-patients at Sappasitthiprasong Hospital, Ubon Ratchathani over 18 years of age with melioidosis were recruited (*Melioid Cohort*) following positive culture of *B*. *pseudomallei* from a clinical specimen, alongside healthy control subjects attending the hospital’s blood donation clinic (*Healthy Control Cohort*) and subjects attending the hospital’s diabetes outpatient clinic (*Diabetes Control Cohort*). For patients who did not attend follow-up, their 28 day survival status was determined by using the hospital mortality records and by telephone.

### Blood samples

Subjects enrolled in the study had 25ml of blood drawn for isolation of peripheral blood mononuclear cells (PBMC), serum and glycated haemoglobin (HbA_1c_) testing for the *Melioid* and *Diabetes Cohorts*. PBMC and plasma were separated by density centrifugation within 3 hours of blood draw and counted with a *Scepter* handheld counter (*Millipore*, *UK*) before use in fresh assays.

### Antigens for assays

Whole heat-inactivated *B*. *pseudomallei* (HIA-Bp) was prepared from two Thai patient isolates 199a and 207a as previously described [29] and used at a dilution of 1:60 (20 μg/ml by Bicinchoninic Acid Assay, *Sigma*) in the ELIspot assay. Phytohemagglutinin (PHA) (final concentration 5 μg/mL) was added to positive control wells. A T-cell epitope pool (*Mabtech*, *AB*, *Sweden*, final concentration 1 μg/mL) was used as control antigens.

### Ex-vivo interferon-γ (IFN-γ) Enzyme-linked immunosorbent spot-forming cell assay (ELIspot) and Indirect Haemagglutination Assay (IHA)

The kinetics and magnitude of the cellular response to whole killed *B*. *pseudomallei* were assessed by fresh *ex-vivo* IFN-γ ELIspot assay. Briefly, 96-well Multiscreen-I plates (*Millipore*, UK) were coated overnight with 1D1K anti-human IFN-γ *(Mabtech*, *AB*, *Sweden)* at 4°C. Fresh PBMCs were added in duplicate wells at 2x10^5^ PBMCs/well and each antigen was added at the optimal concentration. After 18 hours, secreted IFN-γ was detected according to the manufacturer’s instructions *(Mabtech*, *AB*, *Sweden)* and read using a CTL ELIspot reader. Results are expressed as IFN-γ spot-forming cells (SFC) per million PBMC. Background responses in un-stimulated control wells were typically less than 20 spots, and were subtracted from those measured in peptide-stimulated wells. IHA was performed as described previously [29] and a titre of 1:40 or greater was considered positive [30].

### Intracellular staining cytokine assay by flow cytometry

PBMC were stimulated for 18 hours with heat-inactivated *B*. *pseudomallei* (50μg/well) or media. Brefeldin A (Ebioscience, USA) was added at 10 ug/ml and following 4 hours of further incubation, staining for intracellular APC-IFN-γ and for the immune cell surface markers PCP-anti-CD3, FITC-anti-CD4, APCH7-anti-CD8, PE-anti-CD56 and V450-CD14 all BD Biosciences, USA) was performed. Samples were analysed using a MACSQuant Analyzer 10 (Miltenyi Biotec, Germany) with Flowjo software (Treestar Inc, USA). The gating strategy used to identify the cellular source of IFN-γ after stimulation with heat-killed *B*. *pseudomallei* was firstly selection of singlets to exclude aggregates. The lymphocyte populations were then selected for immunophenotyping: double positive CD3+CD4+ for CD4 T cells, double positive CD3+CD8+ for CD8 T cells, double positive CD3+CD56+ for NKT cells and positive CD56 negative CD3 for NK cells. For CD14 positive, cells were gated from singlets. IFN-γ expression levels were then determined by analysis of each cell phenotype. Responses were quantitated for the negative control-subtracted percentage of IFN-γ secreted CD4+ or CD8+ cells from the gated lymphocyte population.

### Data management and statistical analysis

Study clinical data were entered into a password-protected web-based database (*OpenClinica* version 3.1, *LLC and collaborators*, *Waltham*, *MA*, *USA*). The presence of diabetes mellitus in *Melioid Cohort* subjects was defined for analysis as previous diagnosis by a doctor and / or having an HbA_1c_ of ≥ 7% [31]. Subjects in the *Healthy Control Cohort* and *Diabetes Control Cohort* were excluded from the analysis if their IHA titre was greater or equal to 1:40, to allow these cohorts to serve as negative controls. Statistical analysis was performed using Graphpad Prism version 6 (*San Diego*, *CA*, *USA*) and IBM SPSS Statistics for Windows version 22.0 (*Armonk*, *NY*, *USA*). Non-parametric tests were used (Mann Whitney, paired Wilcoxon, Kruskal-Wallis and Spearman’s rank correlation tests) and groups were compared with 2-tailed Fisher’s exact test. To examine the relationship between mortality and the *B*. *pseudomallei*-specific cellular response by ELISpot, a multivariable logistic regression modelling was developed using a purposeful selection method [32] with logarithmic transformation of non-parametric data. A *P* value (2-tailed) of <0.05 was considered significant.

## Results

### Subjects

200 patients with culture-confirmed acute melioidosis were recruited from September 2012 until September 2014 into the *Melioid Cohort* and followed up at 12 weeks (n = 113, 57%) and 52 weeks (n = 94, 47%, follow-up ongoing). On average, participants were enrolled on day 5 after admission (median 5, interquartile range IQR 3 to 6 days). The demographics of the subjects in the study are shown in Table 1. The clinical site of culture of *B*. *pseudomallei* for the *Melioid Cohort* was blood for 105/200 (53%) of cases, with no significant difference between those with and without diabetes. 172/200 (86%) had at least one identifiable risk factor such as diabetes, renal or heart disease, alcohol excess or age greater than 65 years. Antibody titres to *B*. *pseudomallei* were greater than or equal to 40 by indirect haemagglutination assay in 75% (95% CI 68–81) of acute melioidosis patients, consistent with previous reports [1]. 50 subjects were recruited from diabetes outpatients for the *Diabetes Control Cohort*, and 5 were excluded from the analysis for having an IHA ≥40. 50 subjects were recruited from the blood donation clinic for the *Healthy Control Cohort*, and 7 were excluded from the analysis for having an IHA ≥40.

**Table 1:** Subject demographics.

*Cohort*	*N*	*Gender*		*Age In years Mean (range)*	*Diabetes* *	
		*Male n (%)*	*Female n (%)*		*Pre-diagnosed* * *n (%)*	*Study definition* * *n (%)*
**Melioid Cohort all**	**200**	**133 (67%)**	**67 (33%)**	**55 (19–89)**	**117 (59%)**	**134 (67%)**
Melioid Cohort with Diabetes	134	84 (63%)	50 (37%)	53 (19–89)	117 (87%)	134 (100%)
Melioid Cohort no Diabetes	66	49 (74%)	17 (26%)	58 (25–88)	0 (0%)	0 (0%)
**Diabetes Controls Cohort**	**45**	**30 (67%)**	**15 (33%)**	**57 (23–76)**	**45 (100%)**	**45 (100%)**
**Heathy Controls Cohort**	**43**	**26 (60%)**	**17 (40%)**	**48 (33–73)**	**0 (0%)**	**0 (0%)**

The gender, age and diabetes status of subjects in the study are shown. Two thirds (67%) of the patients with acute melioidosis were male, with a mean age of 55 years.

*Diabetes definitions are as follows: Pre-diagnosed = subjects with a previous diagnosis of diabetes mellitus by a doctor. Study definition = subjects with a previous diagnosis of diabetes mellitus by a doctor and / or having an HbA_1c_ of ≥ 7%.

### Diabetes

**Fig 1:**
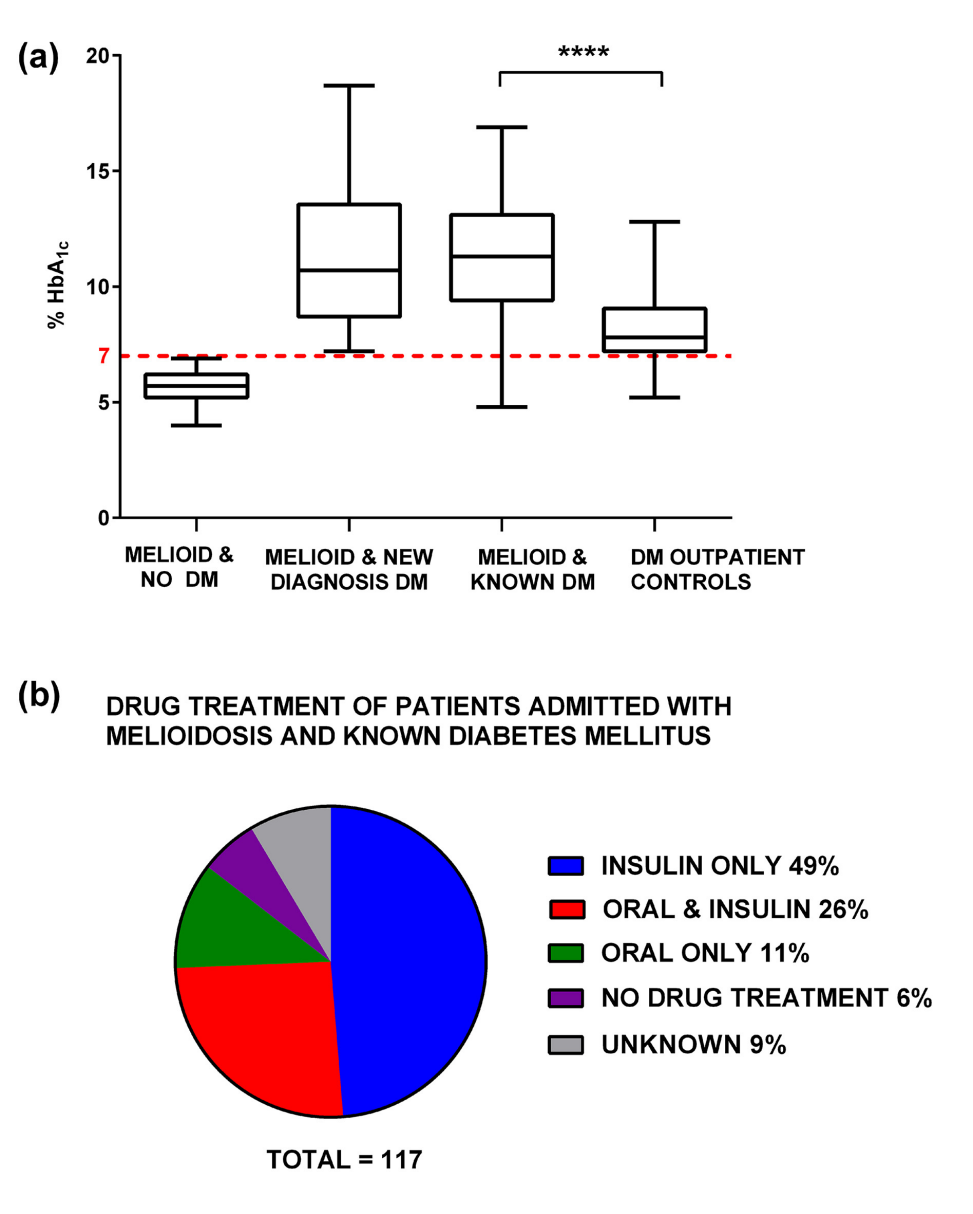
HbA_1c_ for Melioid and Diabetes Cohorts, and treatment received. Fig 1A shows boxplots with Tukey whiskers showing the percentage glycated haemoglobin (HbA_1c_) for subjects in the *Melioid Cohort* (culture-confirmed acute melioidosis) at admission to the study with no diagnosis of Diabetes Mellitus (*Melioid & no DM*), with a new diagnosis of Diabetes Mellitus defined for the study as an HbA_1c_ value of ≥7% (*Melioid & New Diagnosis DM*) and with known Diabetes Mellitus (*Melioid & Known DM*) alongside subjects recruited from diabetes out-patient clinic who are (*DM outpatient controls*).**** *P* < 0.0001 by Mann-Whitney test. Fig 1B shows the drug treatment for diabetes on admission for the 117 patients in the *Melioid Cohort* with previously diagnosed diabetes mellitus.

117 subjects with acute melioidosis were known to have diabetes on admission, with a mean HbA_1c_ of 11.1% (median 11.3, IQR 9.1–13.1). The mean HbA_1c_ of the out-patient diabetic control group was lower at 8.2% (median 7.8, IQR 7.2–9.1) (*P* < 0.001, Fig 1A). Drug information was available for 108/117 of subjects admitted with known diabetes and is shown in Fig 1B. 43 subjects (37%) received at least one oral hypoglycaemic drug including the sulphonylurea glipizide (n = 26), metformin (n = 21), glyburide (n = 4), pioglitazone (n = 4) and glimepiride (n = 1). In addition 17 new diagnoses of diabetes were made on the basis of HbA_1c_ in patients recruited without a previous history of diabetes.

### Clinical outcomes

Mortality data was available for 198/200 patients in the *Melioid Cohort*. 51/198 patients (26%) died within 28 days of admission to hospital despite correct diagnosis and appropriate choice of antibiotic. A further 13 patients died after 28 days (from 29 to 90 days). 28 day mortality was 24% (32/133; 95% CI 17–32) for those with diabetes and 29% (19/65; 95% CI 19–42) for those without diabetes, *P* = 0.49 by Fisher’s exact test). 15/19 (79%) of the non-diabetic subjects who died had at least one identifiable risk factor such as liver or renal disease, excessive use of alcohol or age greater than 65 years. Increasing age was a risk factor for mortality in univariable analysis (*P* = 0.009) but was not an independent risk factor (*P* = 0.29) in the multivariable logistic regression model. Increasing IHA titres were associated with survival (*P* = 0.002) in the univariable analysis, but this was not significant by multivariable analysis and did not contribute to the logistic regression model. Bacteraemia was closely associated with mortality compared to no bacteraemia (*P* < 0.0001) with 39/51 (76%) of the deaths occurring in people who were blood culture positive. The median length of time from admission to hospital until death was 10 days (IQR 5–17), with 35/51 patients taken from hospital to die at home with family. There was no significant relationship between sulphonylurea use in the diabetic melioidosis patients and mortality (*P* = 0.65). Four surviving patients (all with diabetes) had a subsequent diagnosis of melioidosis 2, 5, 6 and 20 months after study entry, defined by positive culture for *B*. *pseudomallei* preceded by negative cultures following standard care including 12 weeks of planned oral follow-on therapy with co-trimoxazole.

### Patients with melioidosis have durable specific T-cell responses to *B*. *pseudomallei*

**Fig 2:**
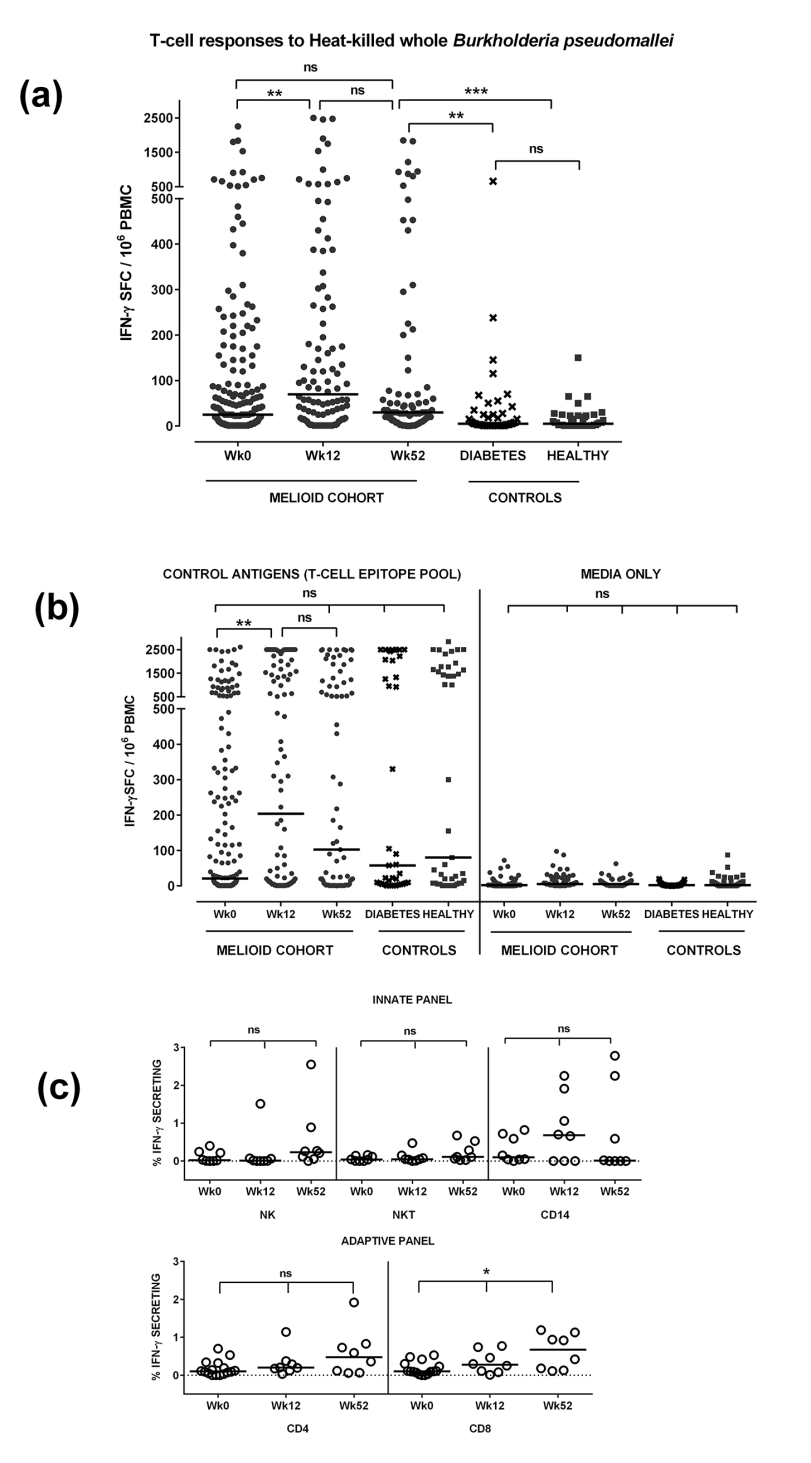
T-cell Responses to Heat killed B. pseudomallei (Fig 2A) to control antigens (Fig 2B) and by cell phenotype (Fig 2C). Peripheral blood mononuclear cells (PBMC) from patients with acute melioidosis (*Melioid Cohort Wk0*) and 12 (*Wk12*) and 52 (*Wk 52*) weeks later were tested alongside PBMC from diabetic outpatients (*Diabetes*) and healthy endemic seronegative control subjects (*Healthy*). Cells were stimulated with heat-inactivated *B*. *pseudomallei* (Fig 2A), a control antigen T-cell epitope pool or media only (Fig 2B) for 18 hours and IFN-γ secreting cells counted and expressed as spot forming cells per million PBMC (SFC/10^6^ PBMC). To characterise the cell phenotype producing IFN-γ, PBMC were incubated with whole *B*. *pseudomallei* for 18 hours and stained for intracellular IFN-γ versus immune cell surface markers (PCP-anti-CD3, FITC-anti-CD4, APCH7-anti-CD8, PE-anti-CD56 and V450-CD14, Fig 2C). Horizontal lines represent medians, ***P<0.001, **P<0.01, *P<0.05, ns = not significant, testing by Kruskal-Wallis with Dunn’s correction for multiple testing.

Cellular immunity to *B*. *pseudomallei* was demonstrated in patients with acute melioidosis (Fig 2A). The mean IFN-γ ELIspot response was 133 SFC per million PBMC (median 25, IQR 3 to 99) at Week 0, 262 (median 70, IQR 15 to 265) at Week 12 and 164 (median 30, IQR 10 to 74) at Week 52. These responses remained elevated at Week 52 compared to the diabetic outpatient controls (mean 37, median 5, IQR 1 to 26, *P* = 0.004) and healthy seronegative controls (mean 15, median 5, IQR 1–23, *P* = 0.0002), whose responses did not differ significantly from each other (*P* = 0.99). Responses to control antigens and media are shown in Fig 2B. Phenotypic analysis demonstrated IFN-γ production by a range of cell phenotypes including CD4, CD8, NK and CD14, with the percentage of IFN-γ producing CD8 cells increasingly significantly from Week 0 to Week 52 (*P* = 0.01, Fig 2C).

### Depressed T-cell responses to B. pseudomallei at admission were associated with higher mortality rates

**Fig 3:**
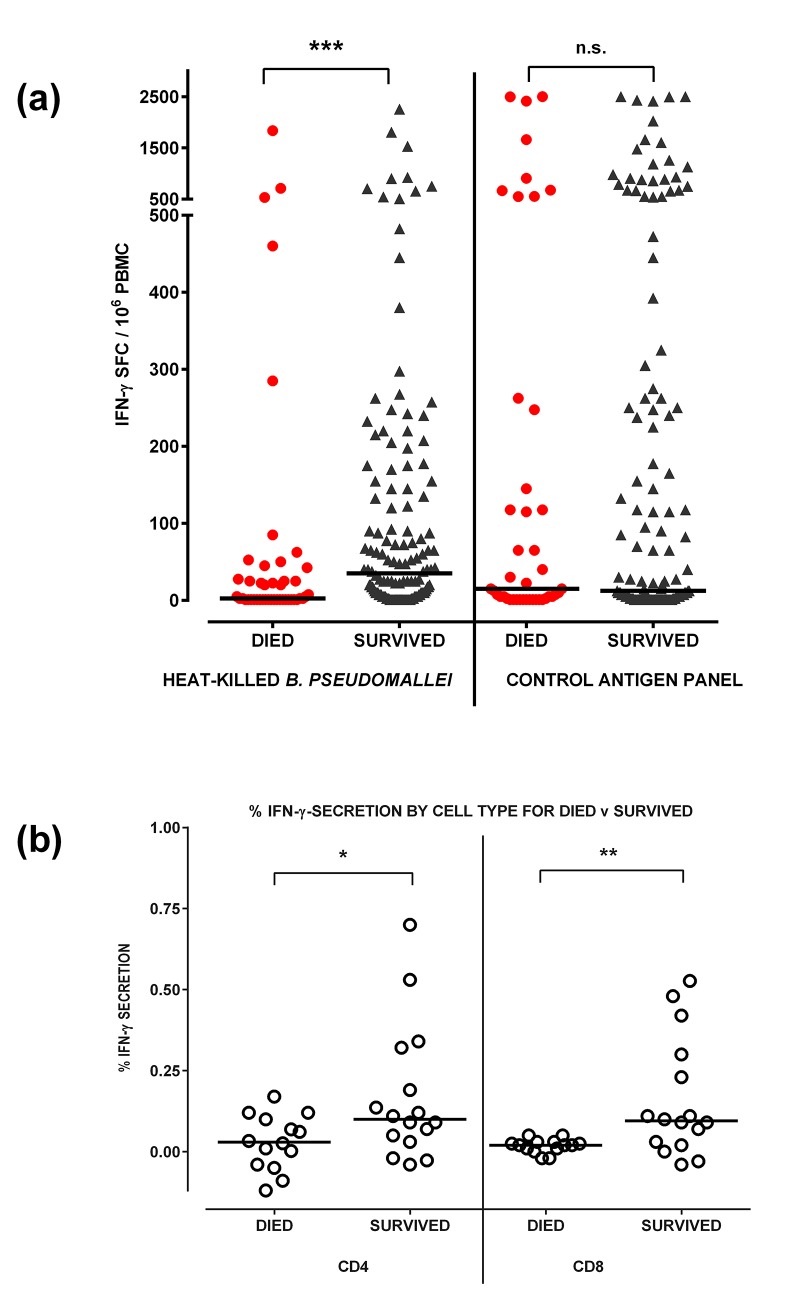
Ex vivo interferon-gamma ELISPOT responses by survival. Peripheral blood mononuclear cells (PBMC) from patients with acute melioidosis at study entry (Week 0) were stimulated with heat-inactivated *B*. *pseudomallei* (Bp), or a control antigen panel for 18 hours and IFN-γ secreting cells counted and expressed as spot forming cells per million (SFC/10^6^) PBMC. Responses were compared for survivors and those who died (Fig 3A). To characterise the cell phenotype producing IFN-γ, PBMC were incubated with whole *B*. *pseudomallei* for 18 hours and stained for intracellular IFN-γ versus immune cell surface markers (PCP-anti-CD3, FITC-anti-CD4 and APCH7-anti-CD8, Fig 3B). Horizontal lines represent medians, ***P<0.001, **P<0.01, *P<0.05, ns = not significant, by logistic regression adjusted for age, diabetes status, renal disease and neutrophil count for responses in Fig 3A and by Mann-Whitney test for Fig 3B.

The mean cellular response in subjects in the *Melioid Cohort* who survived the illness was 142 SFC per million PBMC (median 35 IQR 1–145) compared to 98 (median 3 IQR 1–43) for those who died (Fig 3A). For other antigens tested, there was no significant difference between survivors and non survivors.

The logistic regression model measured the effect of cellular responses to *B*. *pseudomallei*, neutrophil count, age, diabetes status, and the risk factor of known renal disease on the likelihood that participants died within 28 days of admission with acute melioidosis (Table 2). Log_10_ transformed data was used for non-parametric distributions. A tenfold increase in *B*. *pseudomallei*-specific cellular response was associated with reduction by almost half of the likelihood of death (OR 0.56, 95% CI 0.36 to 0.88, *P* = 0.012). Survivors showed an increased frequency of both CD4+ IFN-γ T-cells (*P* = 0.04) and CD8+ IFN-γ T-cells (*P* = 0.009) compared to non-survivors (Fig 3B). The IFN-γ+ CD8+ fraction was 0.05% or less in 14/14 fatal cases studied compared to 5/16 of survivor cases.

**Table 2:** Multivariable analysis for prediction of 28-day mortality in patients with acute melioidosis.

Variable		Adjusted Odds Ratio (95% CI)	*P* value
**Age**		1.0 (0.99–1.1)	0.29
**Diabetes**		0.56 (0.24–1.3)	0.2
**Pre-existing renal disease**		3.9 (1.5–10.5)	0.007
**Bp cell response**		0.56 (0.36–0.88)	0.01
**Neutrophil count / μl**	> 4000–8000	1	1
	≤ 4000	5.8 (1.1–29.6)	0.04
	> 8000–12,000	8.1 (2.1–31.5)	0.002
	> 12,000–20,000	7.5 (1.8–30.6)	0.005
	> 20,000	18.9 (3.1–114.2)	0.001

The table displays the odds ratios for variables studied as predictors of mortality in 200 patients with acute melioidosis in a multivariable logistic regression model. Bp cell response is the log_10_ transformed ELIspot response in fresh peripheral blood mononuclear cells to heat-killed *B*. *pseudomallei*. Neutrophil count represents the peripheral blood neutrophil count with > 4000 to 8000 neutrophils / μl as the comparator group. Odds ratios are adjusted for the other variables included in the model.

Subjects who were bacteraemic had lower cellular responses than subjects who were blood culture negative (*P* = 0.007). The mean cellular response in subjects in the *Melioid Cohort* who were bacteraemic was 99 SFC per million PBMC (median 18 IQR 1–70) compared to 170 (median 46 IQR 5–171) for those who were not bacteraemic.

### In the acute stages of melioidosis depressed cellular responses to B. pseudomallei are observed in patients with diabetes

**Fig 4:**
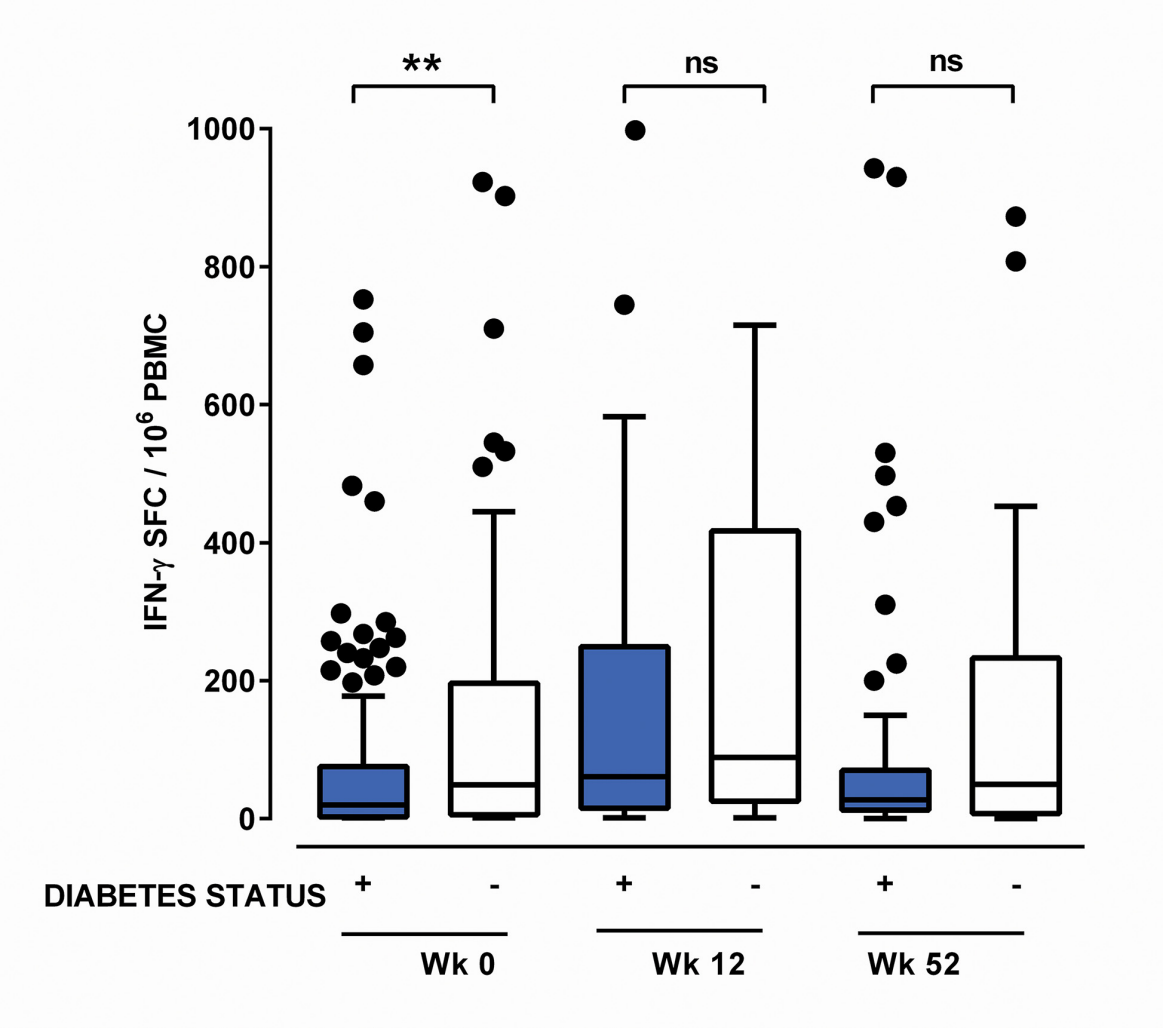
Ex vivo interferon-gamma ELISPOT Responses to Heat killed Burkholderia pseudomallei by diabetes status. Ex vivo IFN-γ ELIspot responses to heat-inactivated *B*. *pseudomallei* are shown for subjects with melioidosis on admission (*Week 0)* and 12 (*Week12*) and 52 (*Week 52*) weeks later according to the presence (+) or absence (-) of a diagnosis of Diabetes Mellitus. Peripheral blood mononuclear cells (PBMC) were stimulated with heat-inactivated *B*. *pseudomallei* for 18 hours and IFN-γ secreting cells counted and expressed as spot forming cells per million PBMC (SFC/10^6^ PBMC). Subjects with diabetes had lower responses to *B*. *pseudomallei* during acute illness (Week 0) compared to subjects with no diabetes. Boxplots with Tukey whiskers are shown with responses greater than 1000 SFC/million PBMC not displayed. ** *P* < 0.01, ns = not significant by Kruskal-Wallis testing with Dunn’s multiple comparisons test.

The cellular response in subjects with acute melioidosis and diabetes was lower than in subjects with acute melioidosis and no diabetes (mean 101 SFC per million PBMC, median 20, IQR 3–76, compared to mean 198, median 49, IQR 6–196), both at Week 0 (*P* = 0.03, Fig 4). There was a non-significant trend for lower responses in subjects with diabetes 12 and 52 weeks later, when subject numbers were lower. There was a non-significant trend towards higher HbA_1c_ being associated with lower cellular responses at Week 0 (Spearman’s r = -0.133, *P* = 0.07) with no clear trend at subsequent timepoints.

### High neutrophil counts during acute melioidosis are associated with lower B. pseudomallei-specific cellular responses in recovery

A higher neutrophil count on admission was also associated with death (median 12,217 neutrophils / μl in fatal cases versus 9,495 neutrophils / μl in survived cases, *P* = 0.002 on univariable analysis). In the multivariable model (Table 2) a “J-shaped curve” effect was seen with higher mortality for both low neutrophil counts (adjusted OR 5.8 [1.1–29.6] for < 4000 neutrophils / μl compared to > 4000 to 8000) and for higher neutrophil counts (adjusted OR 18.9 [3.1–114.2] for > 20,000 neutrophils / μl compared to > 4000 to 8000).

The cellular response to *B*. *pseudomallei* 12 weeks after the illness showed a tight negative association with the peripheral neutrophil count on admission to hospital (*P* < 0.0001, S1 Fig). This relationship between neutrophil count and cellular response was also present at Week 0 (*P* = 0.001), and Week 52 (P = 0.008). No such relationship was seen for the control antigens studied.

## Discussion

The results of this study demonstrate a high mortality for patients with melioidosis in Ubon Ratchathani as previously reported [3]. Work continues in the hospital to audit and improve the “door-to-needle time” for sepsis patients receiving antibiotics but it should be noted that not all patients culture positive for *B*. *pseudomallei* present to hospital with a classical acute sepsis syndrome. The mortality predominantly occurred in patients with bacteraemia rather than contained disease, as previously described [33], with no significant difference in mortality between subjects with diabetes versus no diabetes. Increasing age was a risk factor for death on univariable analysis as previously reported [34]. Age was not an independent risk factor in the multivariable model where cellular immunity and neutrophil count were independent predictors of mortality and may represent age-related immune mechanisms of decline.

Lower *B*. *pseudomallei*-specific T-cell responses were associated with increased mortality. Both CD4+ and CD8+ IFN-γ-producing cells were associated with survival. CD4+ cells, but not CD8+ cells have previously been shown by depletion studies to be important in mediating immunity against experimental melioidosis in a murine model [35].The additional importance of CD8 T-cells in control of melioidosis in humans is supported by our finding that the IFN-γ CD8+ fraction was very low (0.05% or less) in every fatal case whilst the majority of survivors had higher responses. CD8+ cells have been shown to be a source of IFN-γ during primary infection of naïve mice [36] or cell lines [37] via bystander activation but the expansion of *B*. *pseudomallei*-specific IFN-γ CD8+ cell fraction at one year compared to during acute illness supports an adaptive immune role.

The reduced cellular response seen in fatal cases could reflect general immune failure as part of multi-organ failure in the terminal stages. However no such relationship was seen for patients in this study with cellular responses to a control T-cell epitope pool. Establishing the cellular response as having a causal direct role in preventing death, rather than being a marker of outcome is difficult in patient studies, but the correlation demonstrated here supports the hypothesis that T-cell responses are important in the control of *B*. *pseudomallei* infection in humans. Further depletion and adoptive transfer studies in animal models are required to establish causality.

This is the first study to demonstrate the dynamics of the *B*. *pseudomallei*-specific cellular response from acute melioidosis through to follow-up one year later, and the durability of the IFN-γ response is shown. The IFN-γ responses demonstrated in survivors 12 weeks after admission with acute melioidosis are compatible with responses reported in a previous study of T-cell responses to *B*. *pseudomallei* using a longer (42 hour) incubation protocol [27]. The source of the IFN-γ is a combination CD4+ and CD8+ T-cells and Natural Killer cells as previously shown [27] as well as some monocyte production, and we have shown an increase in the *B*. *pseudomallei-*specific IFN-γ CD8+ cell fraction at one year compared to during acute illness. Ongoing studies are evaluating the dynamics of the multifunctional, memory and regulatory cell subsets over time.

The finding of a “J-shaped curve” association between mortality and peripheral blood neutrophil count during acute melioidosis is compatible with the existing literature on the relationship between neutrophils and sepsis [38]. A count in the range of 4000 to 8000 neutrophils/μl was associated with the lowest mortality, and patients with counts above 20,000 neutrophils/μl had an adjusted odd ratio for death by 28 days of 18-fold. Neutrophils play a key role in the host response to *B*. *pseudomallei* [13,39,40] bacteria, but excess activation and inappropriate distribution of neutrophils have been linked to multi-organ failure and poor outcomes in sepsis [38].

There was an inverse relationship between neutrophil count on admission to hospital and the cellular response to *B*. *pseudomallei* on admission, 12 weeks later and 52 weeks later for survivors. Excess neutrophil activation may represent a shift in the development of hematopoietic stem cell progenitors in the bone marrow from lymphoid lineage to myeloid lineage. Although neutrophils are generally believed to support antigen presentation and the development of specific adaptive responses through multiple pro-inflammatory processes, the relationship is complex. Studies have shown inhibition of T-cell responses by neutrophils in bacterial sepsis [41] and by related immature myeloid cells named myeloid derived suppressor cells (MDSCs) [42]. A recent elegant study demonstrated that CD4+ T-cell proliferation and IFN-γ production in response to polyclonal activators is inhibited by *B*. *pseudomallei*-infected neutrophils via Programmed death ligand 1 (PD-L1) [14]. Overall, for generation of adaptive immune responses to *B*. *pseudomallei* excessive neutrophil activation and *B*. *pseudomallei* invasion of neutrophils may be detrimental to the optimal priming environment.

This study shows people with diabetes and acute melioidosis have a depressed cellular response to the bacteria than people without diabetes. This stunted cellular response is of interest because of the increased risk of melioidosis in people with diabetes. The mechanisms of immune dysregulation to intracellular pathogens seen in diabetes are an ongoing focus of research [43]. There are parallels to the relationship between adaptive immunity to tuberculosis and diabetes [44]. In the diabetic mouse model of aerosol challenge with tuberculosis [45], lower IFN-γ levels in the lung were found early in the infection (2 weeks post challenge) compared to non-diabetic mice, but the difference in IFN-γ levels had disappeared by 4 weeks. A temporal delay in the adaptive cellular response to TB was associated with poor early control of intracellular bacteria and ultimately a higher bacterial burden and more inflammation [46]. Our data support a similar relationship in humans and *B*. *pseudomallei*.

Although patients with diabetes had lower cellular responses to *B*. *pseudomallei* while acutely unwell, and lower cellular responses were associated with increased mortality, patients with diabetes did not have an increased mortality in this study, and diabetes was not an independent predictor of death in the multivariable logistic regression model. Previous studies have reported a survival benefit from the diabetes drug glyburide [47] but only one patient in this study took glyburide and use of any sulphonylurea drug was not associated with mortality. Elucidation of the susceptibility of diabetics to this bacterium will have wider implications for prevention of infections in diabetics and those with immune failure.

Patients with melioidosis and underlying known diabetes have poor glycaemic control compared to diabetic out-patients in the region. Healthy patients attending a diabetes outpatient clinic are unlikely to represent the same population because these are people actively engaging with management of their diabetes by attending clinic, whilst the patients in the *Melioid Cohort* have come to medical services because of acute illness. Nevertheless the fact that all but seven patients in the *Melioid Cohort* population with pre-diagnosed diabetes were receiving drug therapy, including insulin in the majority of cases, is evidence of receiving diabetes care. The interaction between diabetes and tuberculosis, another intracellular pathogen, is well known [48], and tuberculosis is reported to worsen glycaemic control [49]. We hypothesise that chronic exposure to *B*. *pseudomallei* may worsen glycaemic control. Because of the impact of rising prevalence of diabetes in Thailand [5] on morbidly and mortality, exacerbation by widespread *B*. *pseudomallei* exposure would have major public health implications. Longitudinal study of the relationship between *B*. *pseudomallei* presence in fields, human infection and glycaemic control are warranted.

### Conclusions

This is the first study to measure the kinetics of a cellular immune response in from acute disease to long-term follow-up, and link results to outcome. Lower cellular responses are associated with increased mortality, supporting a pivotal role for cellular immunity in controlling this bacterium. The development of vaccine strategies that seek to enhance both cell mediated and humoral immunity may be beneficial for successful control of this disease.

## Supporting Information

S1 Fig Relationship between neutrophil count on admission (Week 0) and cellular response to B. pseudomallei on admission, 12 and 52 weeks later. The neutrophil count in cells per microliter for patients acutely unwell with melioidosis (*Melioid Cohort Week 0*) showed a negative correlation with the IFN-γ ELIspot response to *B*. *pseudomallei* (in spot forming cells per million peripheral blood mononuclear cells = SFC/10^6^ PBMC) for the same patients on admission (Week 0) and following recovery 12 and 52 weeks later, calculated by Spearman’s rank correlation coefficient.
(TIF)
