# Supplementary figures and images for: T-Cell Responses Are Associated with Survival in Acute Melioidosis Patients
Source: PLoS Negl Trop Dis. 2015 Oct 23;9(10):e0004152. doi: 10.1371/journal.pntd.0004152 (PMC4619742; doi:10.1371/journal.pntd.0004152)

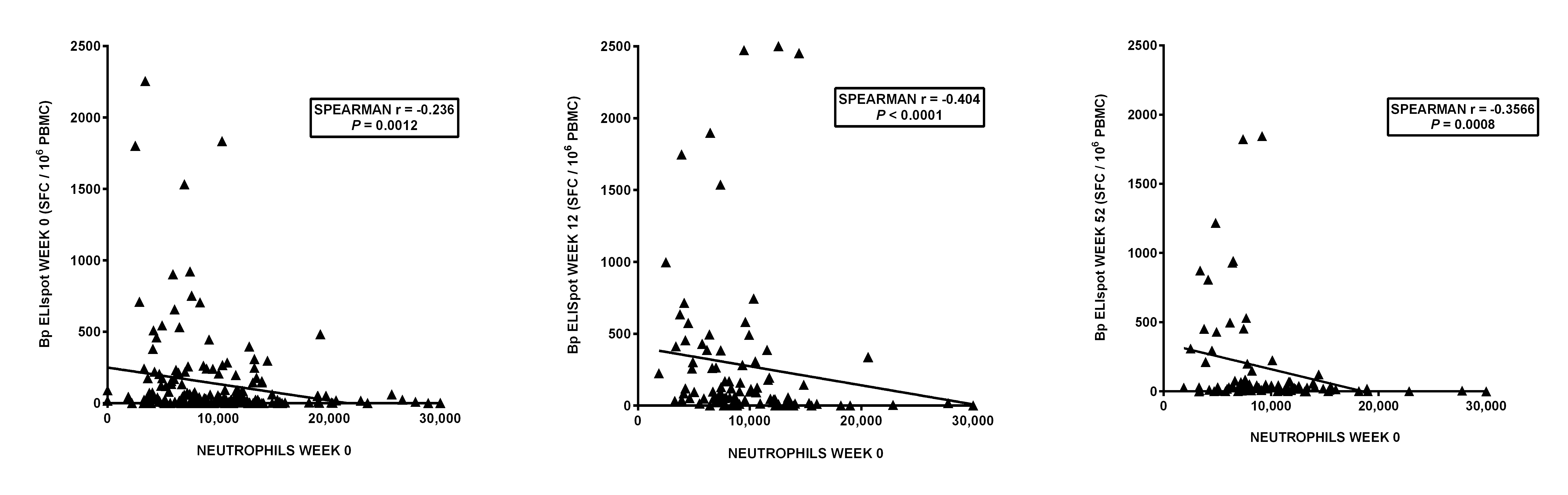

Supplement: S1 Fig — The neutrophil count in cells per microliter for patients acutely unwell with melioidosis (Melioid Cohort Week 0) showed a negative correlation with the IFN-γ ELIspot response to B. pseudomallei (in spot forming cells per million peripheral blood mononuclear cells = SFC/106 PBMC) for the same patients on admission (Week 0) and following recovery 12 and 52 weeks later, calculated by Spearman’s rank correlation coefficient. (TIF) [file pntd.0004152.s001.tif]
